# On Modern Progress in Ophthalmic Medicine and Surgery

**Published:** 1894-05-19

**Authors:** Robert Brudenell Carter

**Affiliations:** Consulting Ophthalmic Surgeon to St. George's Hospital


### On Modern Progress in Ophthalmic Medicine and Surgery.

By Robert Brudenell Carter, F.R.C.S., Consulting Ophthalmic Surgeon to St. George's Hospital.

The introduction of what are collectively called "antiseptic methods" into surgery was more tardy in relation to operations upon the eye than in any other department of the art. The delay was partly due to mechanical difficulties, partly to the character of the antiseptic agents which first came into common use, and partly to familiar experiences which rendered ophthalmic surgeons somewhat less ready than others to yield assent to the whole body of doctrine upon which the practice was supposed to be founded. The ordinary forms of antiseptic dressing were too cumbrous and too heating to be applied to the eye, either readily or with advantage; the contact of carbolic solutions, either in mass or in spray, was often found to be highly irritating to the corneae; and it was quite common, in the extraction of cataract, to witness the entrance of a bubble of air into the anterior chamber, a bubble presumably germ-laden, but which, whether it were coaxed out again or suffered to remain, never appeared to exercise any injurious influence. Nevertheless, although the air of a London bed-room, or even of the operating-theatre of a London hospital seemed to be absolutely innocuous, surgeons were every now and then confronted by cases of delayed healing, or of protracted inflammation, which were manifestly of septic character, and which, in a certain proportion of instances, served absolutely to vitiate the results obtained. The majority of these septic cases have been due, in my opinion, to the introduction into the eye of imperfectly cleaned instruments. On one occasion, now some years ago, when putting together my instruments for an operation, I chanced to let a cystitome fall upon the floor, and, on picking it up, I examined its point with a half-inch doublet to discover whether it had sustained any injury. I was startled to see on one side of the blade a tiny scale of dry lens matter, tinged by blood, the residue of some former operation which had not been removed by the washing customary before the instrument was put away. The scale was too small to be visible to the naked eye, but it was easily detached from the smooth steel by the point of a needle, and it would have been likely to become detached when the cystitome was being used for the division of the capsule. If this had happened a piece of albumen in a state of change would have been left in the anterior chamber, and no surprise could have been felt if septic inflammation had been produced.

From the time of this discovery I have felt the need of exercising the most careful supervision over the absolute cleanliness of instruments, and especially of those such as the iris forceps, the cystitome, and the capsule scissors, which have to be introduced into the anterior chamber. The polished surfaces of the knife and of the iris scissors are easily cleaned by wet cotton wool, and would betray the smallest deposit; but the cystitome and the iris forceps require greater care. The shoulder of the blade of the former, and the space between the teeth of the latter, are places in which the sources of mischief may easily lurk. The best method of cleansing, I think, is by soap and hot water, applied by means of a camel's hair toothbrush and thoroughly rinsed away, after which process and after careful examination with a lens of sufficient power, the instruments should be dipped for a minute or so into absolute alcohol. The knife and scissors, after the more gentle washing called for by the tenderness of their edges, should be dipped into alcohol in the same way, and the surgeon may then feel perfectly secure as far as the risk of septic infection from his instruments is concerned. The air of the room may, I think, be disregarded; but there may be sources of danger in the material used for the manufacture of cocaine wafers, or in the secretions of the conjunctival membrane, as well as in dried scales or secretions clinging to the eyelashes.

In operating for cataract, as in the performance of iridectomy, the surgeon is materially assisted by a small pupil, since such a condition at once deepens the anterior chamber, and renders the position and course of the knife conspicuous against a background of iris. I therefore apply a little eserine, perhaps a quarter or a half of one of Savory and Moore's wafers, about an hour before the operation. Half an hour later the application of cocaine may be commenced, perhaps by two wafers, each containing the fiftieth of a grain, and suffered to remain ten minutes. They should be placed in contact with the surface of the eyeball below the cornea, the lower lid being drawn for the purpose, and the patient looking upwards; but he should be told to look down before the lid is suffered to return to its place, By taking this precaution, the wafers will not be wiped off the eye surface by the rising lid, which otherwise is apt to remove them, and to deposit them upon the eyelashes. After ten minutes, another wafer may be applied, and then another every five minutes until seven have been inserted, by which time the drug will have penetrated to the iris, and the anæsthesia of the structures concerned will be complete. When the application of the wafers is committed to a nurse, the surgeon must be quite sure that she understands the method of inserting them, and that she will be punctual to the times prescribed. Unless these conditions are fulfilled, he may chance to find his patient but imperfectly prepared for the operation. Moreover, as the action of cocaine is only temporary, punctuality on the part of the surgeon himself is equally necessary.

The cocaine will produce its full effect without overcoming that of the eserine which preceded it; and, all being well, the surgeon will find his patient with a small pupil, and with the cornea and conjunctiva insensitive to the contact of instruments. He at once proceeds to wash the eye, so as to remove from it any remaining portions of the gelatine or other material which forms the basis of the wafers, and to remove also anything which may be injurious in the conjunctival secretions. For the purpose of this washing, the best and least irritating antiseptic fluid is, I think, a 15 per cent. solution of Barff's boroglyceride in distilled water, while a morsel of soft and thoroughly aseptic sponge affords the best means of applying it. A receptable should be held by the side of the head, and the patient should look up and down, while the whole surface of the eyeball, the edges and linings of the lids, and the eyelashes, are quickly and effectually cleansed from all adhering matter which could by any possibility be prejudicial.

